# VACCINATION TO PROMOTE HEALTH THROUGHOUT LIFE AS A HEALTHY AGING STRATEGY

**DOI:** 10.1093/geroni/igz038.771

**Published:** 2019-11-08

**Authors:** Leonard Friedland

**Affiliations:** GSK Vaccines, Philadelphia, Pennsylvania, United States

## Abstract

This symposium addresses the role of vaccination to promote healthy aging, the process of developing and maintaining the functional ability that enables wellbeing in older age. Life-span immunization of adults across all age categories can help to reduce morbidity and mortality. Healthy aging is critical for our global society to counter the surge in healthcare costs that is coming as a result of the demographic shift to older age. Immune system function and response to vaccination declines with advancing age. Generating effective immune responses against new infectious disease targets can be difficult in older individuals. Important progress has been made in understanding the mechanisms underlying immunosenescence, the age-related decline of the immune response to infections and vaccinations. Innovative research and the development of new technologies, such as adjuvants, substances that can enhance and shape the immune response to the target antigen(s), has facilitated the development of vaccines specially tailored for adults. This evidence-based approach to the development of innovative vaccines addressing immunosenescence is an important clinically relevant healthy aging strategy to promote health throughout life.

